# Parent‐Adolescent Relationships and Disordered Eating Behavior: Evidence From a Cross‐Sectional Nationwide Study in Brazil

**DOI:** 10.1002/hsr2.71578

**Published:** 2025-12-18

**Authors:** Arthur Eumann Mesas, Renne Rodrigues, Giovana Ribeiro de Souza Favaretto, José Francisco López‐Gil, Vicente Martínez‐Vizcaíno, Alberto Durán González, Estela Jiménez‐López

**Affiliations:** ^1^ Health and Social Research Center Universidad de Castilla‐La Mancha Cuenca Spain; ^2^ Postgraduate Program in Public Health Universidade Estadual de Londrina Londrina Brazil; ^3^ Universidade Federal da Fronteira Sul Chapecó Brazil; ^4^ School of Medicine Universidad Espíritu Santo Samborondón Ecuador; ^5^ Vicerrectoría de Investigación y Posgrado Universidad de Los Lagos Osorno Chile; ^6^ Universidad Autónoma de Chile Facultad de Ciencias de la Salud Talca Chile; ^7^ Department of Psychiatry Hospital Virgen de La Luz Cuenca Spain; ^8^ Centro de Investigación Biomédica en Red de Salud Mental Instituto de Salud Carlos III Madrid Spain

**Keywords:** adolescence, adolescent abuse, Brazil, disordered eating behavior, family relationships, parental neglect

## Abstract

**Background and Aims:**

Disordered eating behaviors (DEBs) in adolescence are influenced by family structure and dynamics, including parental supervision, emotional support, and exposure to neglect or abuse. This study examined the associations between adolescents' relationships with parents or guardians, including indicators of neglect and abuse, and the prevalence of DEBs.

**Methods:**

This cross‐sectional study assessed DEBs and family relationship indicators, including the frequency of shared meals, parental awareness of adolescents' free‐time activities, emotional understanding, and experiences of physical or sexual assault. Data were obtained from the 2019 National School‐Based Health Survey, a representative sample of 95,367 Brazilian students aged 13–17 years. Poisson regression models estimated adjusted prevalence ratios (PRs) and 95% confidence intervals (CIs), stratified by sex.

**Results:**

Fully adjusted analyses revealed that girls were significantly more likely to report DEBs when living with neither parent (PR = 1.34; 95% CI: 1.03–1.75) or never having family meals (PR = 2.36; 95% CI: 1.96–2.84). DEBs were more prevalent in both sexes when their parents did not supervise their school attendance or were unaware of their free‐time activities and when their parents provided limited emotional support. Furthermore, reporting experiencing parental physical aggression or sexual abuse within the past year was also associated with a higher prevalence of DEBs (*p*‐value < 0.05).

**Conclusions:**

The findings suggest that parental neglect is associated with an increased likelihood of DEBs in adolescents and that experiences of parental aggression and sexual abuse may further compound this potential risk. These results underscore the importance of family‐based prevention strategies in addressing adolescent eating disorders.

## 1 Introduction

According to the fifth edition of the Diagnostic and Statistical Manual of Mental Disorders (DSM‐5), eating disorders (EDs) include a range of persistent disordered eating behaviors (DEBs) accompanied by distressing thoughts and emotions [1]. The highest lifetime prevalence of EDs has been reported for binge eating disorder (females: 2.8%, males: 1.0%), bulimia nervosa (females: 1.9%, males: 0.6%), and anorexia nervosa (females: 1.4%, males: 0.2%) [2].

In parallel with the increased incidence of EDs, the prevalence of DEBs has also increased [2, 3, 4]. A recent systematic review estimated that 22.4% of children and adolescents globally experience disordered eating, with higher rates among females (30.0%) than among males (17.0%) [5]. DEBs may precede the onset of clinically diagnosable EDs and are associated with serious psychological, social, physical and mental consequences during adolescence and early adulthood [6, 7, 8]. Therefore, identifying early potential risk factors for DEBs is critical for prevention efforts. Research in primary care settings, particularly among asymptomatic youth, is essential to better define ED‐related conditions and address current knowledge gaps [9].

Multiple risk factors for DEB have been identified, including biological factors (e.g., female sex, later adolescence, genetic predisposition) [10]; sociodemographic and environmental factors (e.g., socioeconomic status, urban living) [11]; behavioral factors (e.g., low physical activity, poor diet quality, and psychoactive substance use) [10, 12]; and psychological factors (e.g., negative body image and poor self‐rated health) [10]. Importantly, experiences of violence victimization, such as bullying, physical aggression and sexual abuse, have also been linked to DEBs [10]. Additionally, the influence of social media on body standards and diet behaviors has emerged as a relevant contributor to DEBs [13].

Family structure and functioning, including cohabitation with parents or guardians and the quality of parent–adolescent relationships, has been increasingly recognized as a key factor in the development and persistence of DEBs during adolescence [13, 14]. Adolescents are more likely to report DEB when exposed to parental criticism or jokes about weight, coercive mealtime dynamics [10], inadequate caregiving [15], and overcontrolling parenting styles [15, 16, 17] or when living in contexts of parental separation, divorce, or broader family dysfunction [18].

Despite the growing body of evidence linking parent‐adolescent relationships to DEBs, few studies have examined multiple relational dimensions simultaneously using nationally representative data. In this sense, the aim of this study was to examine the relationship between adolescents' relationships with their parents or guardians and the prevalence of the specific DEB of “self‐induced vomiting or laxative abuse”. The study proposal was based on three novel contributions. First, analyzing a nationally representative sample enhances generalizability to populations with similar sociodemographic contexts. Second, assessing six dimensions of parent‐adolescent relationships—family structure, frequency of family meals, school supervision, awareness of free‐time activities, emotional support, and physical and sexual abuse—within a single analytical framework provides a more comprehensive understanding of their associations with DEBs. Third, statistical control for sociodemographic, behavioral, and psychosocial confounders was applied to identify which relational aspects are independently associated with DEBs.

## 2 Methods

### 2.1 Study Design

This cross‐sectional study draws on data from the fourth edition of the National School‐Based Health Survey (PeNSE), which was conducted in 2019 in Brazil. The PeNSE is a nationwide initiative supported by the Ministry of Education and forms part of the Brazilian Surveillance of Risk and Protective Factors for Chronic Diseases. All the data were anonymized and are publicly available. The 2019 edition employed a comprehensive sampling strategy that covered the entire national territory, including adolescents aged 13 to 17 years enrolled in public and private schools across both urban and rural areas. Data collection occurred within school settings, where students completed a self‐administered electronic questionnaire using a personal digital assistant device without the presence of a researcher. Further details on the sampling design are available in official documentation [19].

### 2.2 Study Variables

This study is based on secondary analyses of existing surveillance data. Therefore, the included variables were limited to standardized measures that have consistently been collected since 2009 for population‐level monitoring with national representativeness in previous editions of PeNSE.

### 2.3 Dependent Variable

The outcome of interest was the presence of the specific DEB of “self‐induced vomiting or laxative misuse to lose or avoid gaining weight in the past 30 days”. The responses were dichotomized as “yes” or “no”. While this measure does not capture the full spectrum of DEBs, it includes clinically significant compensatory behaviors relevant to public health which align with diagnostic criteria for eating disorders [1]. This measure also enables consistent national monitoring and allows for comparability with other surveillance studies.

### 2.4 Independent Variables

This study examined several indicators of the parent–adolescent relationship. Adolescents reported on their **family structure**, that is, living with their mother, female guardian, father, or male guardian. Based on these responses, four categories were created: living with both parents, living only with the mother, living only with the father, and living with neither parent.

Another item assessed the **frequency of family meals**. This was categorized as daily, sometimes (one to 6 days per week), rarely (less than once per week), or never.

**Supervision of school attendance** was assessed by asking about school absences without parental or guardian permission in the past 30 days. The responses were categorized as “No, did not miss any days without permission” or “Yes, missed one or more days without permission”.

Two additional items were used to evaluate **parental awareness of adolescents' free‐time activities** and **emotional support**. The first item asked how often parents or guardians knew what adolescents were doing in their free time. The second item asked how often parents or guardians understood adolescents' problems and concerns. The response options for both items included never, rarely, sometimes, most of the time, and always. For analytical purposes, rarely and sometimes were grouped, as were most of the time and always.

Finally, two items assessed **parental physical aggression** and **sexual abuse**. The first asked, “In the past 12 months, how many times have you been physically assaulted by your mother, father, or guardian?” Responses were dichotomized into “yes” (one or more times) and “no”. The second item asked “Has anyone ever threatened, intimidated, or forced you to have sexual relations or any other sexual act against your will?” If the respondent answered yes and identified the perpetrator as a parent, stepparent, or guardian (e.g., father, mother, stepfather, or stepmother), the case was classified as parental sexual aggression.

## 3 Covariates

The following covariates were considered: sex assigned at birth (male vs. female); age (13–15 vs. 16–17 years); daily consumption of fruits and vegetables (yes vs. no); number of ultra‐processed foods consumed in the last 24 h (0–3 vs. 4–5 vs. 6–13); total free‐time physical activity (≤ 1 vs. 1–6 vs. ≥ 6 h per week); tobacco smoking in the last 30 days (no vs. yes); alcohol consumption in the last 30 days (no vs. yes); bullying victimization in the last 30 days (no vs. yes); dissatisfaction with body image (no vs. yes); and self‐rated health (optimal vs. suboptimal). Socioeconomic status was assessed using maternal education level, household material goods (e.g., cell phone, computer, and car), and available services (e.g., internet and maid service). We applied principal component analysis (PCA) to these items which creates a weighted composite score in which each indicator contributes according to its ability to discriminate socioeconomic differences in our sample. Finally, the socioeconomic level score was categorized into quartiles. This process was repeated for analyses stratified by sex, considering boys and girls separately.

### 3.1 Statistical Analysis

First, the absolute and relative frequencies of the categorical variables were calculated for the total number of participants, as well as for girls and boys separately. The bivariate associations between each parental or guardian relationship variable and DEB were subsequently assessed via the chi‐square test. Poisson regression models were used to calculate prevalence ratios (PRs) and their 95% confidence intervals (CIs) for the main study associations across the entire sample, stratified by sex. Initially, crude models were adjusted solely for sociodemographic factors (Model 1). These models were then adjusted for lifestyle factors (Model 2) and other covariates (Model 3) to account for potential confounders.

All the statistical analyses were conducted using STATA software, version 15.0 (StataCorp, College Station, TX), considering the parameters of a complex survey design (*svy* commands in STATA). A two‐sided significance level of *α* = 0.05 was used for all tests.

### 3.2 Ethical Aspects

The 2019 PeNSE project was approved by the National Committee of Ethics in Research (CONEP) from the National Health Council (CNS) – Report No. 3.249.268. These institutions regulate and approve human health research, thus further enforcing ethical norms and protecting the confidentiality of the adolescent students interviewed.

## 4 Results

**Figure 1:**
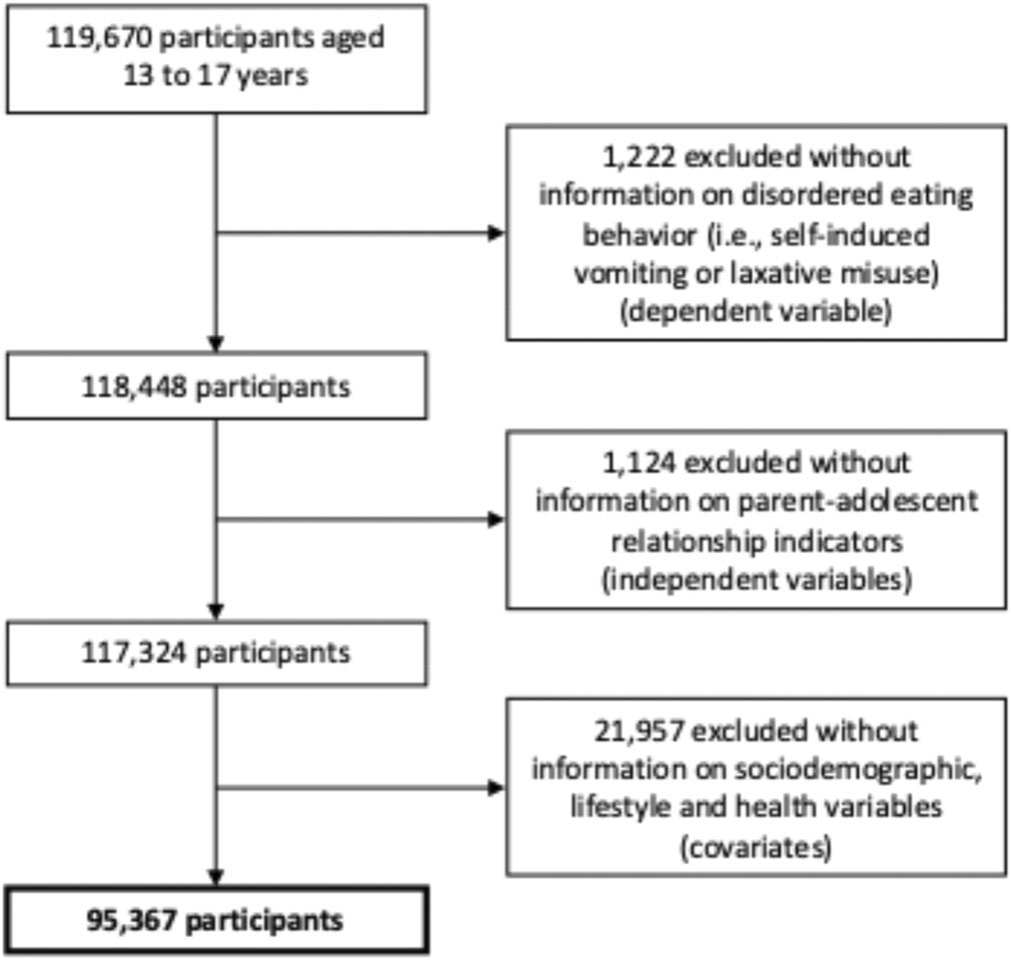
Flow diagram of study participant selection.

As shown in Figure 1, from the total initial population of 119,670 participants aged 13 to 17 years, those without complete information on the DEB (*n* = 1,222), with selected relationships with parents or guardians (*n* = 1,124), and without any of the covariates included in the analyses (*n* = 21,957) were excluded. The majority of participants were excluded because they lacked information regarding ownership of one or more material goods (*n* = 18,408), which prevented the determination of their socioeconomic status. Thus, the final sample for the present analyses comprised 95,367 adolescent students. Considering the proportion of excluded individuals (21.1%), the characteristics of the total original sample were compared with those of the analyzed sample (Supporting Information, Table S1). In general, no relevant differences were observed between the original and analyzed samples.

The characteristics of the studied population are presented in total and separately by sex in Table 1. In summary, the majority of the adolescents were between 13 and 15 years old (66.5%). Differences between the sexes were observed for some lifestyle habits. Specifically, fewer girls reported daily consumption of fruits and vegetables (7.9% vs. 8.7%, respectively) and engaged in more than 6 h of free‐time physical activity per week than boys (13.0% vs. 31.5%, respectively) (Table 1). On the other hand, girls more frequently consumed alcoholic beverages in the last 30 days (29.7% vs. 25.6%, respectively), were victims of bullying in the last 30 days (43.6% vs. 35.9%, respectively), were dissatisfied with their body image (31.9% vs. 13.9%, respectively), and reported suboptimal health (39.3% vs. 21.3%, respectively) compared with boys.

**Table 1:** Characteristics of Brazilian adolescent students overall and by sex.

Characteristic	Total (*N* = 95,367)	Girls (*n* = 49,857)	Boys (*n* = 45,510)
**Age group (years)**, %			
13 to 15	66.5	67.6	65.3
16 to 17	33.5	32.4	34.7
**Socioeconomic status**, %			
1st quartile	25.4	21.3	26.5
2nd quartile	20.9	22.6	23.7
3rd quartile	27.4	31.7	27.3
4th quartile	26.3	24.4	22.6
**Daily consumption of fruits and vegetables**, %			
No	91.7	92.1	91.3
Yes	8.3	7.9	8.7
**Number of UPF consumed in the last 24 h**, %			
0–3	36.1	35.7	36.4
4‐5	34.8	35.0	34.5
6–13	29.1	29.2	29.1
**Total free‐time physical activity (h per week)**, %			
≤ 1	24.9	33.0	15.9
> 1–6	53.3	54.0	52.7
> 6	21.8	13.0	31.5
**Tobacco smoking in the last 30 days**, %			
No	93.6	93.8	93.2
Yes	6.4	6.2	6.8
**Alcohol consumption in the last 30 days**, %			
No	72.3	70.3	74.4
Yes	27.7	29.7	25.6
**Bullying victimization in the last 30 days**, %			
No	60.1	56.4	64.1
Yes	39.9	43.6	35.9
**Dissatisfaction with body image**, %			
No	76.6	68.1	86.1
Yes	23.4	31.9	13.9
**Self‐rated health**, %			
Optimal	69.2	60.7	78.7
Suboptimal	30.8	39.3	21.3

Abbreviation: UPFs, ultra‐processed foods.

As shown in Table 2, the prevalence of DEB was 6.1% in the total population of adolescent students. When disaggregated by sex, DEB was more frequently reported by girls (7.1%) than by boys (4.9%). Significant associations emerged for both the overall sample and for each sex. Specifically, DEB was more likely to be reported by adolescents whose parents supervised their school atendance less frequently, who were aware of what they were doing in their free time, and who were providing emotional support. Additionally, adolescents were more likely to report DEB when they had experienced parental physical aggression or sexual abuse in the past year (*p*‐value < 0.001 for all previous associations) (Table 2). Among girls, DEB was also more prevalent when they reported living with only one parent or with neither of their parents than when they lived with both parents (*p*‐value < 0.001) and when they did not have meals with them daily (*p*‐value < 0.001). This difference was not observed in boys (*p*‐values = 0.823 and 0.174, respectively).

**Table 2:** Frequency of disordered eating behavior (i.e., self‐induced vomiting or laxative misuse) experienced in the last 30 days among Brazilian adolescent students by their relationships with their parents overall and by sex.

Parent‐adolescent relationship indicator	Total			Girls			Boys		
	(*N* = 95,367)			(*n* = 49,857)			(*n* = 45,510)		
	%	DEB, %^a^	*p* value	%	DEB, %^a^	*p* value	%	DEB, %^a^	*p* value
**Total**	100.0	6.2		100.0	7.1		100.0	4.9	
**Family structure**									
Living with both parents	56.1	5.4	< 0.001	54.6	5.8	< 0.001	58.0	4.8	0.823
Living only with the mother	33.3	7.0		35.0	8.4		31.2	5.3	
Living only with the father	4.3	8.0		3.4	11.0		5.2	5.5	
Living with neither parent	6.4	7.6		7.1	9.4		5.6	4.6	
**Frequency of family meals**									
Daily	64.8	5.1	< 0.001	62.9	5.1	< 0.001	66.9	5.0	0.174
Sometimes	14.0	5.6		13.8	7.2		14.3	3.8	
Rarely	14.4	8.8		16.1	11.0		12.5	5.6	
Never	6.8	11.7		7.1	16.2		6.3	6.3	
**Parental supervision of school attendance**									
No	81.1	5.3	< 0.001	82.7	6.2	< 0.001	79.3	4.2	< 0.001
Yes	18.9	9.9		17.3	11.6		20.7	8.0	
**Parental free‐time awareness**									
Always or most of the times	72.1	4.6	< 0.001	74.7	5.7	< 0.001	69.1	3.2	< 0.001
Rarely or sometimes	20.2	8.5		19.3	10.5		21.4	6.2	
Never	7.6	15.0		6.0	13.8		9.5	15.6	
**Parental emotional support**									
Always or most of the times	46.2	4.0	< 0.001	43.0	4.7	< 0.001	49.7	3.2	< 0.001
Rarely or sometimes	37.1	6.6		38.7	7.6		35.3	5.4	
Never	16.7	11.1		18.3	11.8		15.0	9.9	
**Parental physical aggression**									
No	77.6	4.9	< 0.001	77.1	5.5	< 0.001	78.5	4.1	< 0.001
Yes	22.4	10.6		22.9	12.5		21.5	8.2	
**Parental sexual abuse**									
No	99.4	6.0	< 0.001	99.0	7.0	< 0.001	99.7	4.9	< 0.001
Yes	0.6	24.9		1.0	16.9		0.3	52.4	

a Prevalence (%) of the disordered eating behavior (DEB) “self‐induced vomiting or laxative misuse to lose weight or not gain weight” in each category of the relationships with parents. The *p*‐value was obtained via the Chi‐square test.

Multivariate analyses adjusted for the main confounders confirmed the bivariate associations for each sex. As shown in Table 3, the likelihood of girls reporting DEB remained significantly greater when they did not live with both parents, did not share daily meals with them, perceived lower parental awareness of their activities, felt less emotional support from their parents, skipped school without permission, or reported parental physical aggression or sexual abuse in the last year.

**Table 3:** Association between disordered eating behavior (i.e., self‐induced vomiting or laxative misuse) of Brazilian adolescent girls in the last 30 days and their relationships with their parents (*n* = 49,857).

Parent‐adolescent relationship indicator	Crude model	Model 1	Model 2	Model 3
**Family structure**				
Living with both parents	1.00	1.00	1.00	1.00
Living only with the mother	1.45 (1.22, 1.73)*	1.44 (1.21, 1.72)*	1.29 (1.08, 1.54)*	1.22 (1.03, 1.46)*
Living only with the father	1.90 (1.39, 2.58)*	1.88 (1.39, 2.56)*	1.66 (1.22, 2.27)*	1.55 (1.15, 2.10)*
Living with neither parent	1.63 (1.27, 2.09)*	1.60 (1.25, 2.06)*	1.44 (1.11, 1.86)*	1.34 (1.03, 1.75)*
**Frequency of family meals**				
Daily	1.00	1.00	1.00	1.00
Sometimes	1.43 (1.17, 1.75)*	1.45 (1.18, 1.78)*	1.42 (1.16, 1.74)*	1.27 (1.04, 1.54)*
Rarely	2.18 (1.76, 2.71)*	2.18 (1.75, 2.72)*	2.07 (1.67, 2.56)*	1.76 (1.43, 2.16)*
Never	3.20 (2.64, 3.87)*	3.19 (2.64, 3.86)*	2.84 (2.35, 3.42)*	2.36 (1.96, 2.84)*
**Parental supervision of school attendance**				
No	1.00	1.00	1.00	1.00
Yes	1.88 (1.59, 2.22)*	1.86 (1.57, 2.21)*	1.50 (1.25, 1.80)*	1.39 (1.15, 1.67)*
**Parental free‐time awareness**				
Always or most of the times	1.00	1.00	1.00	1.00
Rarely or sometimes	1.85 (1.54, 2.21)*	1.85 (1.55, 2.22)*	1.66 (1.38, 1.99)*	1.51 (1.24, 1.84)*
Never	2.42 (1.89, 3.11)*	2.44 (1.89, 3.15)*	2.10 (1.65, 2.67)*	2.00 (1.56, 2.56)*
**Parental emotional support**				
Always or most of the times	1.00	1.00	1.00	1.00
Rarely or sometimes	1.60 (1.31, 1.95)*	1.59 (1.31, 1.94)*	1.53 (1.25, 1.88)*	1.24 (1.01, 1.53)*
Never	2.48 (1.99, 3.08)*	2.48 (1.99, 3.08)*	2.14 (1.71, 2.69)*	1.59 (1.25, 2.03)*
**Parental physical aggression**				
No	1.00	1.00	1.00	1.00
Yes	2.28 (1.91, 2.71)*	2.31 (1.93, 2.76)*	1.97 (1.63, 2.39)*	1.63 (1.35, 1.98)*
**Parental sexual abuse**				
No	1.00	1.00	1.00	1.00
Yes	2.41 (1.60, 3.63)*	2.40 (1.59, 3.61)*	1.79 (1.23, 2.60)*	1.58 (1.12, 2.23)*

*Note:* The values indicate the prevalence ratio (95% confidence interval) obtained through Poisson regression models. **Model 1**: Adjusted for the following covariates: age group (13–15 years, 16–17 years) and socioeconomic status (categorical, in quartiles). **Model 2**: Model 1 adjusted for daily consumption of fruits and vegetables (no, yes), number of ultra‐processed foods consumed in the last 24 h (0–3, 4–5, 6–13), total free‐time physical activity (≤ 1, > 1 to 6, > 6 h per week), tobacco smoking in the last 30 days (no, yes), and alcohol consumption in the last 30 days (no, yes). **Model 3**: Model 2 was adjusted for bullying victimization in the last 30 days (no, yes), dissatisfaction with body image (no, yes), and self‐rated health (optimal, suboptimal).

* *p*‐value < 0.05.

The corresponding results for boys are presented in Table 4. In contrast to girls, family structure and the frequency of family meals were not associated with DEB in either the unadjusted or adjusted models. However, boys were still more likely to report DEB when they perceived lower parental awareness or emotional support or had experienced parental physical aggression or sexual abuse.

**Table 4:** Association between disordered eating behavior (i.e., self‐induced vomiting or laxative misuse) of Brazilian adolescent boys in the last 30 days and their relationships with their parents (*n* = 45,510).

Parent‐adolescent relationship indicator	Crude model	Model 1	Model 2	Model 3
**Family structure**				
Living with both parents	1.00	1.00	1.00	1.00
Living only with the mother	1.08 (0.85, 1.38)	1.00 (0.79, 1.27)	0.94 (0.75, 1.18)	0.92 (0.73, 1.16)
Living only with the father	1.13 (0.74, 1.73)	1.10 (0.72, 1.69)	0.98 (0.64, 1.52)	0.92 (0.60, 1.43)
Living with neither parent	0.95 (0.64, 1.41)	0.87 (0.58, 1.30)	0.78 (0.53, 1.14)	0.76 (0.52, 1.11)
**Frequency of family meals**				
Daily	1.00	1.00	1.00	1.00
Sometimes	0.75 (0.51, 1.12)	0.87 (0.59, 1.29)	0.84 (0.57, 1.24)	0.79 (0.54, 1.16)
Rarely	1.12 (0.85, 1.48)	1.16 (0.88, 1.54)	1.06 (0.81, 1.39)	0.97 (0.74, 1.27)
Never	1.26 (0.86, 1.84)	1.23 (0.84, 1.80)	1.20 (0.82, 1.73)	1.11 (0.76, 1.61)
**Parental supervision of school attendance**				
No	1.00	1.00	1.00	1.00
Yes	1.92 (1.55, 2.38)*	1.86 (1.49, 2.32)*	1.59 (1.27, 2.00)*	1.52 (1.20, 1.92)*
**Parental free‐time awareness**				
Always or most of the times	1.00	1.00	1.00	1.00
Rarely or sometimes	1.97 (1.56, 2.48)*	1.89 (1.50, 2.38)*	1.74 (1.39, 2.18)*	1.67 (1.33, 2.11)*
Never	4.95 (3.87, 6.33)*	4.49 (3.50, 5.74)*	4.03 (3.15, 5.16)*	3.94 (3.09, 5.03)*
**Parental emotional support**				
Always or most of the times	1.00	1.00	1.00	1.00
Rarely or sometimes	1.67 (1.29, 2.17)*	1.68 (1.30, 2.17)*	1.58 (1.23, 2.03)*	1.47 (1.14, 1.90)*
Never	3.09 (2.32, 4.10)*	2.92 (2.20, 3.87)*	2.71 (2.04, 3.59)*	2.53 (1.89, 3.38)*
**Parental physical aggression**				
No	1.00	1.00	1.00	1.00
Yes	1.99 (1.63, 2.43)*	1.93 (1.58, 2.36)*	1.72 (1.41, 2.10)*	1.54 (1.25, 1.91)*
**Parental sexual abuse**				
No	1.00	1.00	1.00	1.00
Yes	10.80 (7.45, 15.66)*	8.94 (6.09, 13.1)*	7.44 (5.11, 10.8)*	6.68 (4.68, 9.55)*

*Note:* The values indicate the prevalence ratio (95% confidence interval) obtained through Poisson regression models. **Model 1**: Adjusted for the following covariates: age group (13–15 years, 16–17 years) and socioeconomic status (categorical, in quartiles). **Model 2**: Model 1 adjusted for daily consumption of fruits and vegetables (no, yes), number of ultra‐processed foods consumed in the last 24 h (0–3, 4–5, 6–13), total free‐time physical activity (≤ 1, > 1 to 6, > 6 h per week), tobacco smoking in the last 30 days (no, yes), and alcohol consumption in the last 30 days (no, yes). **Model 3**: Model 2 was adjusted for bullying victimization in the last 30 days (no, yes), dissatisfaction with body image (no, yes), and self‐rated health (optimal, suboptimal).

* *p*‐value < 0.05.

The full‐sample results are available in the Supporting Information (Table S2). In the fully adjusted model (Model 3), the association between DEB and family structure was not statistically significant. Nevertheless, all other associations, particularly those related to parental supervision of school atendance, awareness, emotional support, and physical aggression and sexual abuse, were confirmed when girls and boys were analyzed together.

## 5 Discussion

According to the adjusted analyses, certain indicators of the parent–adolescent relationship were associated with DEBs, especially among girls. These factors included family structure (e.g., not living with both parents or guardians) and mealtime routines (e.g., absence of daily family meals). These findings suggest that disruptions in family cohesion and inconsistent parenting may increase the vulnerability of adolescent girls to DEB. Across both sexes, adolescents who reported lower parental awareness of their school attendance and free‐time activities and reduced emotional support from their parents or guardians were more likely to report DEBs. Additionally, those who experienced parental physical or sexual aggression were also more likely to report DEBs. These findings underscore the importance of parental supervision, emotional attunement, and protection from parental violence in preventing eating disorders in adolescents.

The prevalence of DEB was 6.2% in the overall sample, 7.1% in girls and 4.9% in boys. The higher prevalence of DEB in girls is consistent with the state‐of‐the‐art research on this topic [5, 20]. This sex difference may be due to a combination of biological, psychological, and sociocultural factors. Hormonal changes during puberty, body image concerns, and higher levels of perfectionism and anxiety increase the vulnerability of girls [21]. Societal beauty standards, parental and peer influences, and the impact of social media further exacerbate body dissatisfaction and dieting behaviors [22]. Additionally, girls are more likely than boys to experience gendered parenting [23] and weight‐related bullying or teasing from both peers and family [24], which may constitute family‐related factors contributing to the development of DEB.

On the other hand, girls have a greater prevalence of DEB when they do not live with both parents. Evidence suggests that family instability has a greater impact on internalizing problem behaviors (anxiety and depression) in girls than in boys, whereas it has a greater effect on externalizing behaviors (aggression and rule‐breaking) in boys [25]. Girls are particularly vulnerable to emotional distress due to family disharmony [18], which may indirectly increase the risk of DEB. Additionally, separation from mothers (loss of maternal care) or living with mothers who must juggle work and household responsibilities without the support of fathers may increase emotional distress, potentially contributing to an increased risk of DEB [14].

Although some studies have identified mealtime conflict as a source of emotional stress [26], the lack of daily family meals has been suggested as a contributor to the higher prevalence of DEB in girls [27, 28]. This effect may be due to issues related to the process of food introduction and the child's relationship with food [29], as well as the effects that socializing during meals can have on girls, such as improved self‐esteem, an increased sense of purpose, and a positive vision of the future [14, 27].

For both sexes, the prevalence of DEBs was greater when adolescents skipped school without their parents' permission, when parents were less aware of what adolescents did in their free time and when they were less emotionally supportive and less understanding of their children's problems and concerns. These variables reflect how adolescents perceive closeness and empathy in their relationships with their parents and may indicate more harmonious relationships, characterized by greater social support, fewer body and weight‐related conflicts [18, 26], and higher levels of parental care [18]. Furthermore, although there is no specific evidence of this, adolescents' perceptions of parental emotional support may improve their life satisfaction, thereby reducing the pressure to conform to external body standards [30]. The positive influence of parents on the development of children and adolescents has been reinforced when parenting includes affection, discipline, and supervision [27]. Other studies have shown that adolescents who experience low affective expression and low nurturance are at greater risk for developing pathological eating behaviors [31].

Adolescents who reported physical aggression or sexual abuse by their parents or guardians were more likely to experience DEB than those who did not experience such violence. Our results corroborate those of other authors [32]. Previous research has suggested that sexual trauma is a significant risk factor for DEB [33]. One possible explanation is that adolescents may use food to control trauma, block emotions, or express self‐hatred, with more severe trauma correlating with more significant effects. An Italian study revealed a significant association between sexual harassment and DEB in both sexes [34]. Additionally, a case–control study reported that adults with EDs often experienced stressful life events [35]. Although these studies did not specify the aggressor, it is reasonable to assume that family‐based physical or sexual aggression increases the risk of mental disorders, including DEB. Physical victimization, especially when associated with posttraumatic stress disorder, and witnessing or experiencing family violence have also been associated with DEB [36, 37]. Family violence is associated with affective maladjustment and reduced social capital, both of which contribute to DEB [36, 37, 38]. These studies suggest a strong link between family violence, emotional regulation problems, and disordered eating, with DEB potentially serving as a maladaptive coping mechanism.

Some Brazilian sociocultural factors could be particularly important for understanding the associations between parent‐adolescent relationship with DEBs. Brazil is characterized by an appearance culture that is paradoxical in that it simultaneously promotes body diversity while maintaining significant societal pressures regarding physical appearance. This is evidenced by the country ranking among the world leaders in cosmetic surgery procedures [39], as well as by the pervasive exposure to idealized body standards through the media and social networks [40]. Brazil's family structures are diverse and influenced by significant regional and socioeconomic disparities [19]. These disparities often intersect with race, gender, and socioeconomic status, affecting differently adolescent development and health outcomes [41]. These sociocultural factors may increase the risk of developing disordered eating behaviors, especially among adolescents experiencing family dysfunction or inadequate parental support [42].

This study has several limitations that should be considered when interpreting its results. The main limitation is its cross‐sectional design, which prevents causal inferences. Additionally, the results are based on a single measure of DEB (i.e., self‐induced vomiting or laxative misuse in the past 30 days) because this was the only indicator collected in the PeNSE. Therefore, our findings should be interpreted as reflecting associations with these specific DEBs rather than with a more comprehensive assessment of DEB. Moreover, a limited set of adolescent‐reported family structure, mealtime habits, and parenting variables was used, which may not fully capture the complex relationships between parents or guardians and adolescent DEB. It is important to note that all study variables were self‐reported by the adolescents. Therefore, underreporting of DEBs and biased reporting of their relationships with their parents cannot be ruled out. Another limitation is the potential for residual confounding, even with many covariates included in the analysis. Unmeasured factors such as personality or mood disorders, social media use, or unrelated trauma and stress may also influence outcomes.

Nevertheless, this study has notable strengths. The findings are based on a nationally representative sample of students aged 13 to 17 years. Unlike many earlier studies, this analysis accounted for sex as a potential moderating factor and controlled for age and various sociodemographic, lifestyle and health‐related factors.

In conclusion, our findings suggest that for adolescent girls, living with both parents and sharing daily family meals are associated with a higher prevalence of DEB. Adolescents of both sexes who perceive their parents as attentive and supportive are also less likely to develop DEB. These results highlight the value of promoting family meals to strengthen parent–child relationships, particularly through dialog and trust. They also emphasize the importance of consistent parental involvement in adolescents' overall behavior, not just their eating habits, to prevent DEB. Finally, increased vulnerability is evident among girls living with one parent or with neither parent nor among adolescents of both sexes who have experienced physical aggression or sexual abuse by their parents or guardians. These adolescents should receive social health support, including interventions to prevent DEB, which can lead to EDs and severe outcomes such as malnutrition, suicidal ideation, suicide attempts, and death.

## Author Contributions

**Arthur Eumann Mesas:** conceptualization (lead), formal analysis (lead), writing – original draft preparation (lead), writing – review and editing (equal). **Renne Rodrigues:** writing – review and editing (equal). **Giovana Ribeiro de Souza Favaretto:** conceptualization, writing – original draft preparation (equal), writing – review and editing (equal). **José Francisco López‐Gil:** writing – review and editing (equal). **Vicente Martínez‐Vizcaíno:** writing – review and editing (equal). **Estela Jiménez‐López:** writing – original draft preparation (equal), writing – review and editing (equal). **Alberto Durán González:** conceptualization, writing – original draft preparation (equal), writing – review and editing (equal). All authors have read and approved the final version of the manuscript. **Arthur Eumann Mesas** had full access to all of the data in this study and takes complete responsibility for the integrity of the data and the accuracy of the data analysis.

## Ethics Statement

All procedures followed were in accordance with the ethical standards of the responsible committee on human experimentation (institutional and national) and with the Helsinki Declaration of 1975, as revised in 2000. The 2019 PeNSE project was submitted and approved by the National Committee of Ethics in Research (CONEP) from the National Health Council (CNS) – Report No. 3.249.268 (April 8, 2019). These institutions regulate and approve health research involving human beings, thus further enforcing ethical principles and safeguarding the confidentiality of the information of the adolescents interviewed. Informed consent was obtained from all adolescent students involved in the study and from their parents or guardians.

## Conflicts of Interest

The authors declare no conflicts of interest.

## 1 Transparency Statement

Arthur Eumann Mesas affirms that this manuscript is an honest, accurate, and transparent account of the study being reported; that no important aspects of the study have been omitted; and that any discrepancies from the study as planned have been explained.

## Supporting information


**Table S1.** Characteristics of the total sample and analyzed sample of adolescent students, Brazil, 2019. **Table S2.** Association between disordered eating behavior (i.e., self‐induced vomiting or laxative misuse) of Brazilian adolescent students in the last 30 days and their relationships with their parents (N=95,367).

## Data Availability

All of the data used in this study have been anonymized and are publicly available at https://www.ibge.gov.br/estatisticas/sociais/educacao/9134-pesquisa-nacional-de-saude-doescolar.html?=&t=resultados (accessed on 9 October 2023).
