# Supplementary material for: Parent‐Adolescent Relationships and Disordered Eating Behavior: Evidence From a Cross‐Sectional Nationwide Study in Brazil
Source: Health Sci Rep. 2025 Dec 18;8(12):e71578. doi: 10.1002/hsr2.71578 (PMC12712865; doi:10.1002/hsr2.71578)
Supplement: Supplementary file 1 — Table S1. Characteristics of the total sample and analyzed sample of adolescent students, Brazil, 2019. Table S2. Association between disordered eating behavior (i.e., self‐induced vomiting or laxative misuse) of Brazilian adolescent students in the last 30 days and their relationships with their parents (N=95,367). [file HSR2-8-e71578-s001.docx]

**SUPPORTING INFORMATION**

**Parent-Adolescent Relationship and Disordered Eating Behavior:**

**Evidence from a Nationwide Study in Brazil**

**Table S1.** Characteristics of the total sample and analyzed sample of adolescent students, Brazil, 2019.

| **Characteristic** | **Total**  **sample, %**  (N=119,670)* | **Analyzed sample, %**  (n=95,367) |
| --- | --- | --- |
| Sex (girls) | 50.4 | 52.3 |
| Age group (13 to 15 years) | 66.5 | 66.5 |
| Socioeconomic status (1^st^ quartile) | 25.5 | 25.4 |
| Daily consumption of fruits and vegetables (yes) | 8.2 | 8.3 |
| Number of ultra-processed foods consumed in the last 24 h (0-3) | 37.1 | 36.1 |
| Total free-time physical activity (>6 hours/week) | 21.4 | 21.8 |
| Tobacco smoking in the last 30 days (yes) | 6.7 | 6.4 |
| Alcohol consumption in the last 30 days (yes) | 27.0 | 27.7 |
| Bullying victimization in the last 30 days (yes) | 39.3 | 39.9 |
| Dissatisfaction with body image (yes) | 22.5 | 23.4 |
| Self-rated health (suboptimal) | 29.9 | 30.8 |
| Family structure (living with both parents) | 55.5 | 56.1 |
| Frequency of family meals (daily) | 65.0 | 64.8 |
| Parental supervision of school attendance (no) | 19.4 | 18.9 |
| Parental free-time awareness (always or most of the times) | 40.8 | 41.7 |
| Parental emotional support (always) | 24.2 | 24.2 |
| Parental physical aggression in the last year (yes) | 21.6 | 22.4 |
| Parental sexual abuse in the last year (yes) | 0.7 | 0.6 |
| Self-induced vomiting or laxative misuse (yes) | 6.2 | 6.2 |

* The percentages are calculated based on the total number of adolescent students and the available data for each variable.

**Table S2**. Association between disordered eating behavior (i.e., self-induced vomiting or laxative misuse) of Brazilian adolescent students in the last 30 days and their relationships with their parents (N=95,367).

| **Parent-adolescent relationship indicator** | **Crude Model** | **Model 1** | **Model 2** | **Model 3** |
| --- | --- | --- | --- | --- |
| **Family structure** |  |  |  |  |
| Living with both parents | 1.00 | 1.00 | 1.00 | 1.00 |
| Living only with the mother | 1.31 (1.14, 1.52)* | 1.25 (1.08, 1.44)* | 1.13 (0.98, 1.30) | 1.09 (0.94, 1.25) |
| Living only with the father | 1.49 (1.15, 1.94)* | 1.52 (1.16, 1.98)* | 1.35 (1.03, 1.77)* | 1.25 (0.95, 1.64) |
| Living with neither parent | 1.42 (1.14, 1.76)* | 1.32 (1.06, 1.64)* | 1.18 (0.95, 1.47) | 1.12 (0.89, 1.41) |
| **Frequency of family meals** |  |  |  |  |
| Daily | 1.00 | 1.00 | 1.00 | 1.00 |
| Sometimes | 1.11 (0.92, 1.33) | 1.18 (0.97, 1.42) | 1.15 (0.95, 1.39) | 1.04 (0.87, 1.25) |
| Rarely | 1.72 (1.46, 2.04)* | 1.69 (1.44, 2.00)* | 1.58 (1.35, 1.85)* | 1.38 (1.18, 1.60)* |
| Never | 2.29 (1.90, 2.76)* | 2.25 (1.87, 2.70)* | 2.05 (1.72, 2.44)* | 1.77 (1.49, 2.10)* |
| **Parental supervision of school attendance** |  |  |  |  |
| No | 1.00 | 1.00 | 1.00 | 1.00 |
| Yes | 1.88 (1.63, 2.16)* | 1.89 (1.64, 2.18)* | 1.57 (1.35, 1.82)* | 1.47 (1.26, 1.71)* |
| **Parental free-time awareness** |  |  |  |  |
| Always or most of the times | 1.00 | 1.00 | 1.00 | 1.00 |
| Rarely or sometimes | 1.87 (1.61, 2.17)* | 1.87 (1.61, 2.17)* | 1.68 (1.45, 1.95)* | 1.57 (1.36, 1.83)* |
| Never | 3.29 (2.71, 4.00)* | 3.36 (2.76, 4.09)* | 2.97 (2.46, 3.59)* | 2.87 (2.39, 3.43)* |
| **Parental emotional support** |  |  |  |  |
| Always or most of the times | 1.00 | 1.00 | 1.00 | 1.00 |
| Rarely or sometimes | 1.67 (1.41, 1.96)* | 1.64 (1.39, 1.92)* | 1.56 (1.33, 1.84)* | 1.35 (1.14, 1.60)* |
| Never | 2.78 (2.34, 3.31)* | 2.64 (2.22, 3.15)* | 2.36 (1.97, 2.84)* | 1.93 (1.60, 2.33)* |
| **Parental physical aggression** |  |  |  |  |
| No | 1.00 | 1.00 | 1.00 | 1.00 |
| Yes | 2.18 (1.89, 2.51)* | 2.18 (1.88, 2.52)* | 1.90 (1.63, 2.21)* | 1.62 (1.39, 1.89)* |
| **Parental physical aggression** |  |  |  |  |
| No | 1.00 | 1.00 | 1.00 | 1.00 |
| Yes | 4.13 (3.02, 5.65)* | 3.75 (2.69, 5.22)* | 2.98 (2.14, 4.15)* | 2.61 (1.93, 3.54)* |

The values indicate the prevalence ratio (95% confidence interval) obtained through Poisson regression models. **Model 1**: Adjusted for the following covariates: age group (13-15, 16-17 years) and socioeconomic status (categorical, in quartiles). **Model 2**: Model 1 adjusted for daily consumption of fruits and vegetables (no, yes), number of ultra-processed foods consumed in the last 24 h (0-3, 4-5, 6-13), total free-time physical activity (≤1, >1 to 6, >6 h per week), tobacco smoking in the last 30 days (no, yes), and alcohol consumption in the last 30 days (no, yes). **Model 3**: Model 2 adjusted for bullying victimization in the last 30 days (no, yes), dissatisfaction with body image (no, yes), and self-rated health (optimal, suboptimal). **p*-value <0.05.
